# Effective Synergy of Sorafenib and Nutrient Shortage in Inducing Melanoma Cell Death through Energy Stress

**DOI:** 10.3390/cells9030640

**Published:** 2020-03-06

**Authors:** Fernanda Antunes, Gustavo J. S. Pereira, Renata F. Saito, Marcus V. Buri, Mara Gagliardi, Claudia Bincoletto, Roger Chammas, Gian Maria Fimia, Mauro Piacentini, Marco Corazzari, Soraya Soubhi Smaili

**Affiliations:** 1 Department of Pharmacology, Federal University of São Paulo, Paulista School of Medicine, São Paulo 04021-001, Brazil; fernanda_antunes84@hotmail.com (F.A.); jspereira.gustavo@gmail.com (G.J.S.P.); claudia.bincoletto25@gmail.com (C.B.); soraya.smaili23@gmail.com (S.S.S.); 2 Center for Translational Research in Oncology, Department of Radiology and Oncology, Faculty of Medicine of the University of São Paulo and Cancer Institute of the State of São Paulo, São Paulo 04021-001, Brazil; renata.saito@hc.fm.usp.br (R.F.S.); rchammas@usp.br (R.C.); 3 Department of Molecular Biology, Federal University of São Paulo, Paulista School of Medicine, São Paulo 04021-001, Brazil; marcus.buri@gmail.com; 4 Department of Health Sciences (DISS), University of Piemonte Orientale, 28100 Novara, Italy; mara.gagliardi@uniupo.it; 5 Center for Translational Research on Autoimmune and Allergic Disease (CAAD), 28100 Novara, Italy; 6 Department of Epidemiology and Preclinical Research, National Institute for Infectious Diseases IRCCS ‘Lazzaro Spallanzani’, 00149 Rome, Italy; gianmaria.fimia@inmi.it (G.M.F.); mauro.piacentini@uniuroma2.it (M.P.); 7 Department of Molecular Medicine, University of Rome La Sapienza, 00185 Rome, Italy; 8 Institute of Cytology of the Russian Academy of Sciences, 199034 Saint Petersburg, Russia; 9 Department of Health Sciences and Interdisciplinary Research Center of Autoimmune Diseases (IRCAD), University of Piemonte Orientale, 28100 Novara, Italy

**Keywords:** apoptosis, energy stress, melanoma, autophagy, sorafenib

## Abstract

Skin melanoma is one of the most aggressive and difficult-to-treat human malignancies, characterized by poor survival rates, thus requiring urgent novel therapeutic approaches. Although metabolic reprogramming has represented so far, a cancer hallmark, accumulating data indicate a high plasticity of cancer cells in modulating cellular metabolism to adapt to a heterogeneous and continuously changing microenvironment, suggesting a novel therapeutic approach for dietary manipulation in cancer therapy. To this aim, we exposed melanoma cells to combined nutrient-restriction/sorafenib. Results indicate that cell death was efficiently induced, with apoptosis representing the prominent feature. In contrast, autophagy was blocked in the final stage by this treatment, similarly to chloroquine, which also enhanced melanoma cell sensitization to combined treatment. Energy stress was evidenced by associated treatment with mitochondrial dysfunction and glycolysis impairment, suggesting metabolic stress determining melanoma cell death. A reduction of tumor growth after cycles of intermittent fasting together with sorafenib treatment was also observed in vivo, reinforcing that the nutrient shortage can potentiate anti-melanoma therapy. Our findings showed that the restriction of nutrients by intermittent fasting potentiates the effects of sorafenib due to the modulation of cellular metabolism, suggesting that it is possible to harness the energy of cancer cells for the treatment of melanoma.

## 1. Introduction

Metabolic reprogramming is a hallmark of tumor cells in which the glycolytic process is boosted compared to mitochondrial metabolism to produce energy (‘Warburg effect’) [1,2]. This is also particularly important for cancer cell lines due to their microenvironment represented by rich medium usually supplemented with high glucose. However, this concept is changing in recent years, due to accumulating data showing high plasticity of cancer cells in modulating cellular metabolism to adapt to a heterogeneous and continuously changing microenvironment, at least in solid tumors [3,4,5,6]. Therefore, the real contribution of mitochondria to cancer cell survival is still unclear and under deep investigation. Beyond its crucial role in tumor development and growth, glucose addiction is also implicated in tumor chemoresistance, inhibiting cancer therapy efficacy and thus negatively impacting on patient survival rates [7]. For metastatic melanoma for example, BRAF activating mutations (~60% of oncogenic mutations) [8,9], in addition to contributing to uncontrolled proliferation, invasion, autophagy deregulation, and resistance to therapy [8,10], are also associated with melanoma metabolic changes as increase of glucose capture and high rate of glycolysis [11] contribute to poor therapeutic outcome. Although specific BRAF inhibitors have been developed in the last decade, their therapeutic failure as single agents or associated with other anticancer drugs, boosted the research toward new and alternative therapeutic approaches. Of note, new immunotherapy regimens, based on PD1 and/or CTLA4 antibodies, are under clinical trial experimentation with promising results, although showing mild/severe side effects, such as autoimmune-related diseases, that could limit their broad clinical application [12,13]. Therefore, new therapeutic strategies are still urgently needed to treat metastatic melanoma patients. Recent studies demonstrated that dietary manipulations such as caloric restriction and intermittent fasting can inhibit tumor growth improving the anticancer therapy in several human cancers [14,15,16,17]. Although definitive data are still missing, the positive effect of nutrient restriction on chemotherapeutic regimens seems to be related to the metabolic stress imposed on cancer cells. We have recently shown that melanoma cells are sensitive to this therapeutic design based on nutrient shortage coupled with cisplatin treatment, in in vitro studies [18,19]. Here we extend this concept by using an intermittent fasting regimen coupled with sorafenib treatment. Sorafenib is a multi-kinase inhibitor (included BRAF) and Food and Drug Administration (FDA) approved anticancer drug used to treat renal cell carcinoma and advanced hepatocellular carcinoma [20,21]. Thus, here we reinforce the use of new therapeutic strategies based on regimens of intermittent fasting coupled with anticancer drugs as an emerging attractive therapeutic approach to apply to resistant and aggressive tumors, such as human skin melanoma.

## 2. Materials and Methods

### 2.1. Cell Lines and Treatments

Cell identity was confirmed by short tandem repeat analysis (STR) and the DSMZ Online STR Analysis [22], and mycoplasma testing was routinely performed each month by using the Venor^®^GeM Classic (Minerva-BiolAbs, Berlin, Germany). Human melanoma BRAF^WT^ (CHL-1, C8161) and oncogenic BRAF^V600E^ (A375, A2058, SK Mel 05, and SK Mel 28) were cultured in DMEM high glucose supplemented with 10% fetal bovine serum (Gibco) and 1% penicillin/streptomycin solution (Gibco) (complete medium), at 37 °C under 5% CO_2_ [23]. Cells were treated with Earle’s balanced salt solution (EBSS; Sigma-Aldrich, St. Louis, MO, USA), multi-tyrosine kinase inhibitor sorafenib (SOR; Santa Cruz Biotechnology, Dallas, TX, USA) 10 μM; caspase pan-inhibitor Z-VAD-FMK (Santa Cruz Biotechnology, Dallas, TX, USA) 50 μM; necroptosis inhibitor necrostatin-1 (NEC; Sigma-Aldrich, St. Louis, MO, USA) 50 μM; glycolysis inhibitor 2-deoxi-glucose (2-DG; Sigma-Aldrich, St. Louis, MO, USA) 10 mM; lysosomal acidification inhibitor chloroquine (CQ; Sigma-Aldrich, St. Louis, MO, USA) 25 μM; proteasome inhibitor Mg132 (Sigma-Aldrich, St. Louis, MO, USA) 5 μM, mitochondrial depolarization agent carbonyl cyanide 4-(trifluoromethoxy) phenylhydrazone (FCCP; Sigma-Aldrich, St. Louis, MO, USA) 50 μM, and hydrogen peroxide (H_2_O_2_; Sigma-Aldrich, St. Louis, MO, USA) 50 mM.

### 2.2. Cell Death Evaluation

Cell death was evaluated as previously described [24]. Briefly, 1 × 10^5^ cells were plated onto 24-well microplates and treated as indicated. Then, cells were fixed with cold ethanol (50% in Phosphate buffer saline (PBS)), pelleted and resuspended in RNAse (50 μg/mL in PBS), incubated at 37 °C for 15 min followed by propidium iodide (PI—25 μg/mL in PBS) staining. The percentage of sub-G1 cells was evaluated by flow cytometry. Using a FACS Calibur cytometer (Becton-Dickinson, Mountain View, CA, USA) 10,000 events were acquired in FL2 channel, and the sub-G1 percentage was analyzed using FlowJo™ Software.

### 2.3. Western Blotting Analysis

Protein extraction was performed by using NP-40 lysis buffer (50 mM Tris-HCl pH 7.4, 1% NP-40, 150 mM NaCl, 5 mM EDTA), supplemented with protease inhibitor cocktail (Sigma-Aldrich) plus phosphatase inhibitors (10 mM sodium fluoride, 1 mM sodium orthovanadate, and 1 mM sodium molybdate; Sigma-Aldrich, St. Louis, MO, USA). Lysates were incubated at 4 °C for 30 min, then centrifuged at 4 °C for 10 min at 12,000× *g*. Protein concentration was determined using a Bradford assay (Bio-Rad, Hercules, CA, USA), in which 20 μg of total proteins was resolved by using SDS-PAGE gels (Life Technologies) and electroblotted onto PVDF or nitrocellulose membranes (Millipore, Burlington, MA, USA). Blots were incubated with primary antibodies resuspended in 5% non-fat dry milk (Sigma-Aldrich, St. Louis, MO, USA) in PBS plus 0.1% Tween-20 overnight at 4 °C. Primary antibodies were: anti-PARP (1:1000, Cell Signaling, Danvers, MA, USA); anti-Mcl-1 (1:1000, Cell Signaling, Danvers, MA, USA), anti-LC3B (1:1000, Cell Signaling, Danvers, MA, USA), anti-ATG5 (1:1000, Cell Signaling, Danvers, MA, USA); anti-AMPK (1:1000, Cell Signaling, Danvers, MA, USA); anti-p-AMPK (Thr172) (1:1000, Cell Signaling, Danvers, MA, USA); anti-p70S6K1 (1:1000, Cell Signaling, Danvers, MA, USA), anti-p-p70S6K1 (Thr 389; 1:1000, Cell Signaling, Danvers, MA, USA), anti-GAPDH (1:5000, Sigma-Aldrich, St. Louis, MO, USA), and anti-Tubulin (1:5000, Sigma-Aldrich, St. Louis, MO, USA). Detection was achieved using a horseradish peroxidase-conjugate secondary antibody (Jackson Immunoresearch, Ely, UK; 1:10,000 in 5% non-fat dry milk in PBS plus 0.1% Tween-20), visualized with ECL (GE Healthcare, Chicago, IL, USA) and images were recorded by using a ChemiDoc imaging platform (Uvitec, Cambdrige, UK) and analyzed by Uvitec Alliance software.

### 2.4. Lentiviral Generation and Transduction

Co-transfection of lentiviral vectors (shRNA-pLKO ATG5, or scrambled sequence, 10 μg; Sigma-Aldrich), vesicular stomatitis virus G protein expression plasmid (5 μg) and psPAX2 plasmid (carrying *gag*, *pol*, and *rev* genes) was performed using 293T packaging cell line, by a calcium phosphate protocol [23]. Supernatants with lentiviral particles were harvested 48 h later and supplemented with 4 μg/mL of polybrene. These supernatants were used to transduce target cells [24].

### 2.5. Retrovirus Generation and Transduction

Co-transfection of retroviral vectors (15 μg; GFP-mCherry-LC3) and vesicular stomatitis virus G protein expression plasmid (5 μg) was performed by using 293 gp/bsr cell line and calcium phosphate protocol [23]. Supernatant with retroviral particles was harvested 48 h later and supplemented with 4 μg/mL of polybrene. The supernatants were used to transduce target cells.

### 2.6. Confocal Microscopy for Autophagy Evaluation

GFP-mCherry-LC3 transduced cells as previous described were plated on 13 mm glass coverslip at 1 × 10^5^ density and after adhesion were starved by using EBSS (STV) or treated with CQ 25 μM, SOR 10 μM and SOR + STV for 6 h. Cells were fixed with 4% paraformaldehyde in PBS and detected in Zeiss LSM 780 Confocal Microscopy (Carl Zeiss, Oberkochen, Germany). Images were analyzed by ImageJ software.

### 2.7. ΔΨm Assessment

Briefly, 1 × 10^5^ cells were treated for 2 h, as indicated and cells harvested, pelleted, resuspended in TMRE (50 nM in PBS), and incubated at 37 °C for 15 min in the dark. Using a FACS Calibur cytometer (Becton-Dickinson, Franklin Lakes, NJ, USA) 10,000 events were acquired. Data analysis was performed using FlowJo software. Alternatively, 3.5 × 10^5^ cells were platted on 40 mm glass coverslip and 24 h later were stained with TMRE (50 mM in complete medium) followed by real-time confocal microscopy. Cells were maintained under TMRE (50 nM) in complete medium or EBSS (STV) and were imaged every 5 s. After the establishment of a basal line, cells were stimulated with SOR 10 μM and followed for 30 min. Images were detected in Zeiss LSM 780 Confocal Microscopy and analyzed by the software ZEN lite (Carl Zeiss, Oberkochen, Germany).

### 2.8. Real-Time PCR Analysis

Total RNA was extracted by using TRIzol™ reagent (Invitrogen, Carlsbad, CA, USA) as recommended by the supplier. cDNA synthesis was performed using a reverse transcription kit (Promega, Madison, WI, USA) according to the manufacturer’s recommendations. Quantitative PCR reactions were performed by using a Rotor-Gene 6000 (Corbett Research Ltd., Saffron Walden, UK) thermocycler. Maxima SYBR Green/ROX qPCR Master Mix (2X) (Thermo Fisher Scientific, Waltham MA, USA) was used to produce fluorescently labeled PCR products. Primer sets for PGC1α amplicons (forward: 5′-GAGCGCCGTGTGATTTAT-3′ and reverse: 5′-CATCATCCCGCAGATTTACT-3′) were designed using Primer-Express 1.0 software (Roche, Basel, Swiss). L34 (forward: 5′-GTCCCGAACCCCTGGTAATAGA-3′ and reverse 5′-GGCCCTGCTGACATGTTTCTT-3′) mRNA level was used as an internal control and results were expressed as previously described [25].

### 2.9. In Vivo Experiments and Tissue Processing

The animal model experiments were carried out in accordance with the guidelines for animal experimentation determined by the Medical School of University of São Paulo (FMUSP) and conducted in accordance with the Institutional Animal Ethics Committee (IAEC). Six- to eight-week-old male athymic NOD/SCID mice were housed in a 12 h light/12 h dark schedule at 24 ± 2 °C temperature, 50% ± 10% relative humidity under pathogen-free conditions. Mice were subcutaneously injected with SK Mel 28 cells (2.5 × 10^6^ cells/mouse) in order to initiate tumor growth. On the 25th day after inoculation, mice were randomly divided into four groups (*n* = 6), namely CTR (controls), IF (intermitted fasting), SOR (sorafenib), and SOR + IF. CTR and SOR animals had free access to food and water, while IF and SOR + IF animals had 24 h cycles of no food intake (fasting) and free access to water intercalated with 24 h cycles of free access to food and water. All animals were subjected to oral gavage with vehicle (DMSO 6%-PBS) (CTR and IF) or Sorafenib (40 mg/kg-DMSO 6%-PBS) (SOR and SOR + IF) for five consecutive days every week. Tumor size was measured on alternate days, and tumor volume was calculated by the formula long diameter (mm) × short diameter^2^ (mm^2^) × 0.5236. All animals were weighed every day. On the 39th day after tumor cell inoculation, the animals were euthanized, the tumors collected, and snap-frozen in liquid nitrogen for western blotting analysis. For tissue extraction, NP-40 lysis buffer (50 mM Tris-HCl pH 7.4, 1% NP-40, 150 mM NaCl, 5 mM EDTA) was added, supplemented with a protease inhibitor cocktail (Sigma-Aldrich, St. Louis, MO, USA) plus phosphatase inhibitors (10 mM sodium fluoride, 1 mM sodium orthovanadate, and 1 mM sodium molybdate) (Sigma-Aldrich, St. Louis, MO, USA) and homogenized in Ultra-Turrax^®^ for 3 min on ice. Tissue extracts were then centrifuged at 12,000× *g* at 4 °C and supernatants collected for determination of protein concentration by Bradford assay (Bio-Rad, Hercules, CA, USA). The western blotting analysis proceeded as described above.

### 2.10. Statistics

All values are represented as the mean ± SD. Significance was evaluated by ANOVA one-way followed by Bonferroni test for multiple comparisons among control and treatments. ANOVA two-way followed by Bonferroni post-test was used for group analysis. Differences were considered significant with *p* < 0.05. For in vitro studies, at least three independent experiments were conducted to warrant that the results were representative. For animal study, considering a significance level * of 5%, a variation coefficient between 15% and 20%, and the effect of 20% it was necessary to have 6 animals/group for statistic evaluation in one in vivo experiment [26,27].

## 3. Results

### 3.1. Fasting Consistently Enhances Sorafenib-Induced Cell Death in Human Melanoma Cells

**Figure 1:**
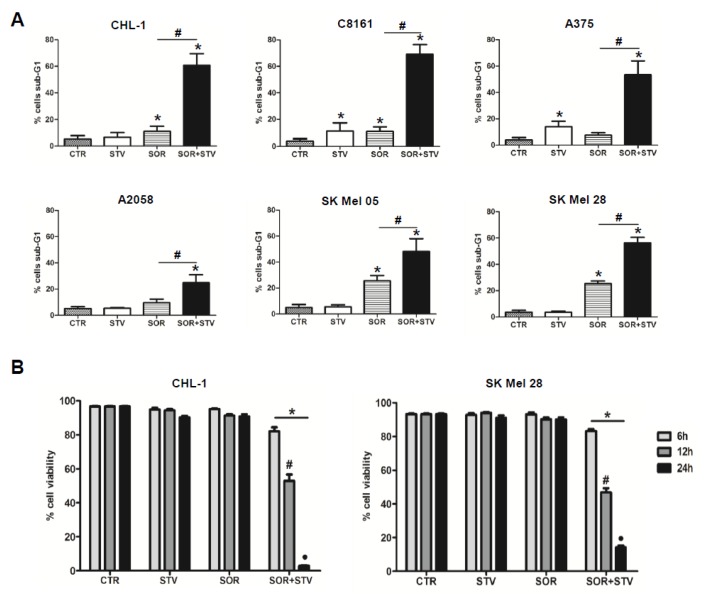
Combined exposure to sorafenib and starvation induces cell death in human melanoma cells. (**A**) Cell death evaluation (percentage of sub-G1 population) of PI-stained BRAF^WT^ (CHL-1 and C8161) and oncogenic BRAF^V600E^ (A375, A2058, SK Mel 05, and SK Mel 28) melanoma cell lines untreated (CTR) or exposed to EBSS (STV), sorafenib (SOR 10 mM) and SOR + STV for 24 h. (Histograms represent mean ± SD, *n* = 3) * *p* < 0.0001 compared to control cells; # *p* < 0.0001 compared to sorafenib-treated cells. One-way ANOVA, Bonferroni post-test. (**B**) Cell viability was analyzed in CHL-1 and SK Mel 28 cells at 6, 12, and 24 h of STV, SOR, and SOR + STV post treatment. (Histograms represent mean ± SD) * *p* < 0.0001 compared to control cells. # *p* < 0.0001 compared to 6h; • *p* < 0.0001 compared to 6 and 12 h. Two-way ANOVA, Bonferroni post-test.

It is widely manifest that short-term starvation (nutrient restriction) sensitizes or resensitizes many cancers to chemotherapeutic treatments [15,28], providing a potential powerful therapeutic strategy to overcome resistance of different tumor types, such as human skin melanoma [18]. Indeed, due to the urgent need of an effective and durable therapeutic regimen to treat patients affected by skin melanoma and to increase the overall patient survival rate, we decided to explore this opportunity. To this aim, a panel of human melanoma cell lines (BRAF^WT^ and BRAFV^600E^) were submitted to EBSS (starvation, STV) or treated with sorafenib under normal (SOR, 10 μM) and starvation conditions (SOR + STV) for 24 h. The subsequent cell death induction was evaluated in all cell lines by measuring the percentage of sub-G1 populations of propidium iodide (PI)-stained cells, by flow cytometry. Data depicted in Figure 1A clearly show that most cell lines were resistant to STV or SOR treatments individually while all of them were significantly sensitive to combined (SOR + STV) exposure. Then, we decided to use CHL-1 and SK Mel 28 cell lines (a BRAF^WT^ and oncogenic BRAF^V600E^, respectively) as models in the next experiments. These cells were then treated with SOR or cultured in EBSS (STV) alone or in combination (SOR + STV) and cell viability was evaluated at 6, 12, or 24 h post treatment. Results confirmed the decrease of cell viability in both cell lines exposed to SOR in combination with STV (Figure 1B) in a time-dependent manner, further sustaining the efficacy of combined nutrient restriction and chemotherapeutic treatment as potential therapeutic regimen design, compared to individual treatments.

### 3.2. Apoptosis Is Involved in Combined Sorafenib/Nutrient Restriction-Induced Cell Death

To elucidate the molecular mechanism(s) responsible for melanoma cell death induction/execution under combined SOR/STV treatment, we evaluated the presence/expression of key apoptotic markers such as PARP cleavage and Mcl-1 degradation, in both cell lines treated 2, 4, or 6 h with SOR or STV alone or in combination by western blotting analysis.

**Figure 2:**
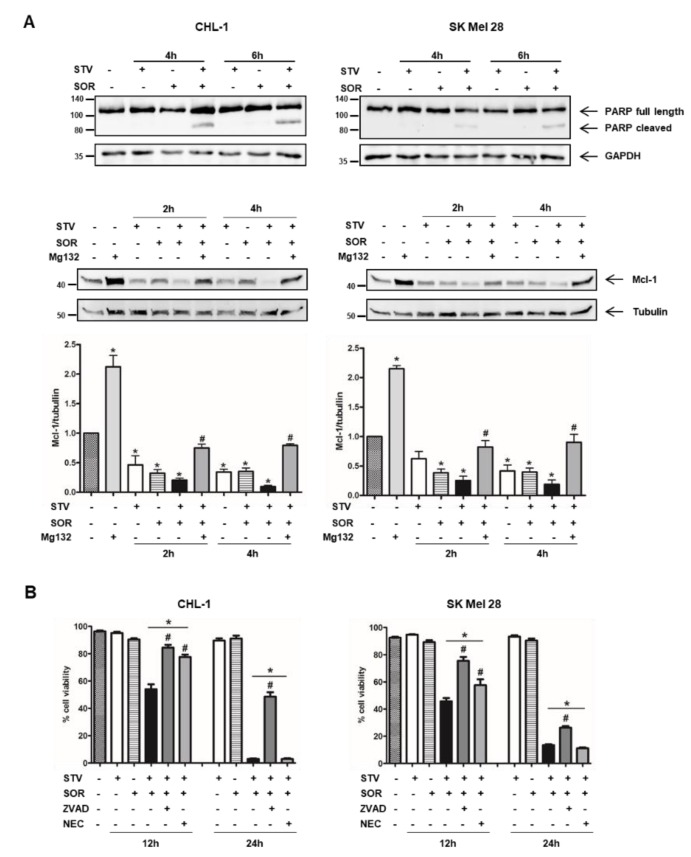
Cell death elicited by SOR + STV on melanoma cells has apoptotic features. (**A**) Representative immunoblots of PARP cleavage and Mcl-1 degradation (apoptotic markers) analyzed by western blotting in CHL-1 and SK Mel 28 cells untreated or treated as indicated. GAPDH or tubulin were used as loading controls. (Histograms represent mean ± SD) * *p* < 0.0001 compared to untreated cells; # *p* < 0.0001 compared to Mg132 absence. One-way ANOVA, Bonferroni post-test. (**B**) Cell viability was also evaluated in both cell lines after 12 or 24 h of STV, SOR, SOR + STV, Z-VAD-FMK, or necrostatin-1 (NEC), as indicated. (Histograms represent mean ± SD) * *p* < 0.0001 compared to control cells. # *p* < 0.0001 compared to SOR + STV. One-way ANOVA, Bonferroni post-test.

Our results highlighted an early activation of caspases, key mediators of the apoptotic program and responsible for PARP cleavage, in both cell lines but restricted to the combined SOR + STV regimen (Figure 2A, upper panels). The expected lower sensitivity of SK Mel 28 cells compared to CHL-1 is also evident, due to the presence of oncogenic BRAF^V600E^ conferring less sensitivity to apoptotic stimuli [8,29,30,31], evidenced by a less pronounced PARP cleavage efficiency at both 4 and 6 h post treatment compared to CHL-1. The involvement of the apoptotic pathway is also highlighted by the dramatic Mcl-1 downregulation in both cell lines exposed to SOR + STV (Figure 2A, middle/bottom panels), compared to control or each individual treatment (SOR or STV), both known to affect Mcl-1 expression [32,33,34,35]. Finally, to further support the involvement of apoptosis, we exposed both cell lines to STV in combination with SOR (SOR + STV), in the presence or absence of the pan-caspase inhibitor Z-VAD-FMK (ZVAD) or necrosis inhibitor necrostatin-1 (NEC), and cell viability was evaluated after 24 h. As shown in Figure 2B, the presence of ZVAD, but not necrostatin-1, was able to protect both cell lines from SOR + STV-induced cell death, although a minor effect was observed in SK Mel 28, suggesting the involvement of a caspase-independent pathway activated by sorafenib in this cell line, as previously suggested by Lachaier [36]. Collectively these data indicate that apoptosis is involved in the cell death pathway stimulated by combined sorafenib/nutrient shortage in melanoma cells.

### 3.3. Combined Nutrient Shortage and Sorafenib Induces Metabolic Stress Resulting in Cell Death

**Figure 3:**
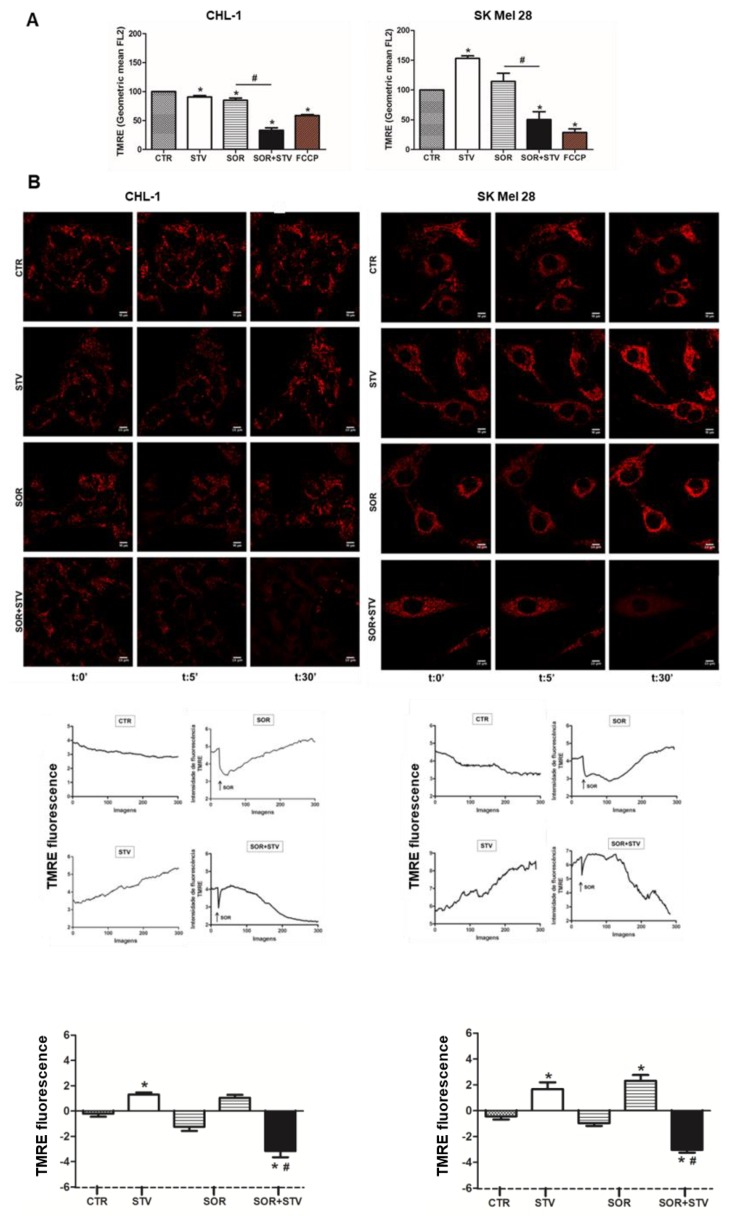
Combined exposure to sorafenib and starvation compromises mitochondrial function. (**A**) Mitochondrial membrane potential (MMP, ΔΨ) was evaluated by flow cytometric analysis of TMRE-stained CHL-1 or SK Mel 28 cells after STV, SOR, or SOR + STV exposure (2 h). FCCP was used as positive control. (**B**) Representative florescence images of three time points (t = 0′, t = 5′, and t = 30′) and time lapse analysis were performed by real-time confocal microscopy to evaluate ΔΨ in CHL-1 or SK Mel 28 TMRE-stained cells treated as indicated. Histograms represent the quantitative analysis of TMRE fluorescence variation after 30′ in both cell lines treated as indicated. (Images are representative of three independent experiments; histograms represent mean ± SD; *n* = 3) * *p* < 0.005 compared to control cells. # *p* < 0.0001 compared to sorafenib.

**Figure 4:**
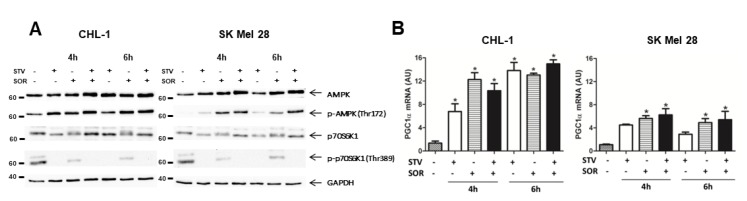
Combined exposure to sorafenib and starvation stimulates biogenesis in human melanoma cells. (**A**) The activity of the AMPK and mTOR signaling pathways were evaluated by measuring the phosphorylation status of AMPK (p-AMPK on Thr172) or the mTOR target p70S6K1 (p-p70S6K1 on Thr389) in CHL-1 and SK Mel 28 cells treated or untreated (4 or 6 h) as indicated, by western blotting analysis. Total AMPK, p70S6K1, and GAPDH were used as internal/loading controls (*n* = 3). (**B**) The expression of PGC1α was evaluated by qRT-PCR in the same experimental condition described in A. (Histograms represent mean ± SD; *n* = 3) * *p* < 0.005 compared to control cells.

Cell transformation and tumor development determine a well-known metabolic reprogramming with cancer cells preferring glycolysis to mitochondrial catabolism to produce ATP (Warburg effect) [1,2]. Therefore, we asked whether combined sorafenib and nutrient shortage-induced cell death was the result of metabolic stress. To this aim, we evaluated the mitochondrial activity in cells exposed to EBSS or sorafenib alone or in combination, by measuring alterations in the mitochondrial transmembrane potential (MTP) by flow cytometry. Although SOR is known to target mitochondrial proteins (OXPHOS) [37,38], data in Figure 3A, show the MTP was not (CHL-1) or slightly (SK Mel 28) altered in both cell lines in presence of SOR or EBSS alone, while a consistent and early depolarization was observed in both cell lines concomitantly exposed for 2 h to SOR + EBSS. These data possibly indicate that the 2 h treatment with SOR we used in these experiments was not sufficient, per se, to consistently disrupt mitochondrial functions—that possibly cope the mild stress condition through the fission/fusion mechanism [39]—while concomitant treatment was efficient in inducing a consistent mitochondrial stress. These data were confirmed by a time course analysis (0, 5, and 30 min) of MTP (TMRE-staining) in cells exposed to EBSS (STV), SOR, SOR + STV, in which images were acquired by confocal microscope and analyzed by ImageJ software. Data indicate a clear mitochondrial depolarization in both cell lines concomitantly exposed to STV and SOR but not in individual exposure (Figure 3B). However, the analysis of ROS (reactive oxygen species) production in the same experimental conditions revealed an increase in ROS generation in SK Mel 28 cells but not in CHL-1, possibly indicating a more efficient antioxidant system of the latter cell line compared to the former but, more importantly, that ROS production is not the main cell death mediator in SOR + STV exposed cells (Supplementary Figure S1). Then, we analyzed the signaling pathways typically activated under metabolic stress conditions. To this aim, cells exposed for 4 or 6 h to STV, SOR, or STV + SOR were lysed and total protein extracts were subjected to western blotting analysis. Data reported in Figure 4A (upper panels) show the previously described AMPK activation (P-AMPK) under EBSS (STV) [5] or SOR [40] exposure, that was even more evident in cells concomitantly exposed to both treatments, to face the dramatic increased of AMP/ATP ratio resulting from latter experimental condition (SOR + STV). The energy stress condition under STV + SOR is confirmed by concomitantly observed mTOR inhibition, evidenced by disappearance of its target P-p70S6K1 (Figure 4A, bottom panels), typical of persistent AMPK activation [41]. Cell’s response to metabolic stress was also evidenced by analyzing the expression of PGC1α, the key transcription factor strongly activated by conditions causing energy limitation [42]. Indeed, measuring the mRNA levels of this TF (transcriptional factor) revealed its upregulation in both cell lines under STV or SOR treatment (at both 4 and 6 h) as expected, due to cell’s energy limitation and demand under both treatments, condition that was confirmed under both SOR and STV exposure (Figure 4B). These results were further confirmed by measuring the cell viability of cells (CHL-1 and SK Mel 28) exposed to SOR or STV alone or in combination (SOR + STV) in the presence or absence of the glycolytic inhibitor 2-DG (2-deoxy-D-glucose). Data reported in Supplementary Figure S2 show indeed an enhanced cytotoxic effect of combined SOR and STV in both cell lines cultivated in the presence of 2-DG, due to efficient inhibition of glycolysis, compared to limited inhibition due to low glucose content of EBSS (STV).

### 3.4. Autophagy Blockade Is Involved in the Signaling Pathway Stimulated by Combined Sorafenib and Nutrient Shortage

Nutrient shortage and cellular stresses are typical inducers of autophagy, an intracellular catabolic process responsible for the removal of damaged or unwanted cellular structures/organelles and the production of energy to sustain cell survival [9,43]. However, although promptly induced under stress conditions, this process remains active for a limited time, due to a negative feedback loop responsible for early stabilization and subsequent degradation of key autophagic components [44,45]. On the other hand, although highly debated, prolonged or deregulated autophagy might be responsible for or participate in cell death execution [46]. To evaluate the potential contribution of autophagy to the signaling pathway stimulated by combined sorafenib and nutrient shortage regimen in melanoma cells, both CHL-1 and SK Mel 28 were exposed to SOR or STV alone or in combination, in the presence or absence of the downstream autophagic inhibitor chloroquine (CQ).

**Figure 5:**
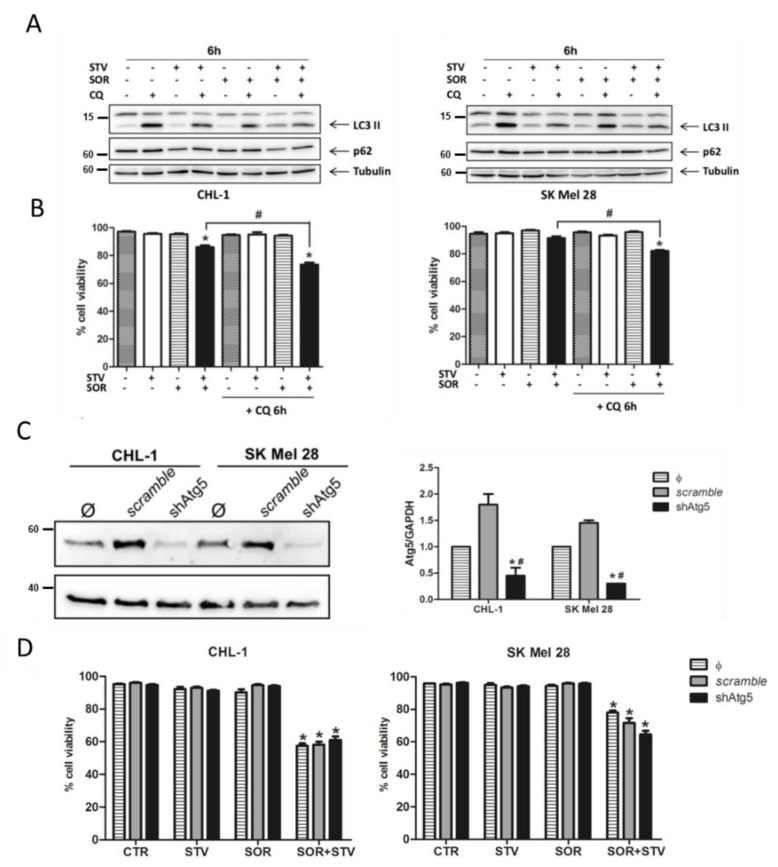
Late-stage autophagy blockage is involved in SOR + STV-induced cell death of melanoma cells. (**A**) Autophagy was evaluated by western blotting analysis, by measuring LC3 conversion and p62 degradation in CHL-1 and SK Mel 28 cells exposed 6 h to EBSS (STV), SOR [10 mM] or SOR + STV, in presence or absence of the autophagic inhibitor chloroquine (CQ 25 mM). Tubulin was used as loading control (Images are representative of three independent experiments). (**B**) Cell viability of CHL-1 and SK Mel 28 was also performed in the same experimental conditions reported in A. (Histograms represents mean ± SD; *n* = 3) * *p* < 0.005 compared to control cells. # *p* < 0.0001 absence vs. presence of CQ. (**C**) ATG5 expression was abrogated in both CHL-1 and SK Mel 28 by transducing cells with lentiviral particles carrying a specific shRNA sequence for Atg5 (shAtg5) or a scrambled sequence (scramble). Atg5 protein levels were evaluated by western blotting analysis (left panel) and a densitometric analysis (Atg5 normalized by GAPDH) was also performed (right panel). GAPDH was used as loading control. (Images are representative of three independent experiments; histograms represent mean ± SD, *n* = 3) * *p* < 0.0001 compared to not transduced cells (Φ); # *p* < 0.0001 compared to scramble cells. (**D**) Cell viability was evaluated by flow cytometry in both control cells and shAtg5 cells under STV, SOR, or combined treatment (SOR + STV), at 6 h post treatment. (Histograms represent mean ± SD; *n* = 3) * *p* < 0.0001 compared to untreated cells (CTR).

The protein levels of p62 and LC3-II were then evaluated by western blotting analysis, 6 h post treatment to detect the autophagic flux (Figure 5A and Supplementary Figure S3). These data show no significant decrease of p62 protein levels in any condition, suggesting that ubiquitinated proteins/organelles are not targeted by autophagy in these conditions, but a very slight increase of LC3-II in the SOR + STV condition was observed even without chloroquine, indicating a possible block in late-stage autophagy. Similar results were obtained by confocal analysis of autophagosome formation in both cell lines transduced with a vector encoding for a recombinant GFP-mCherry-LC3 protein and exposed to similar experimental condition reported above (Supplementary Figure S4). Cell viability was also evaluated in cells exposed to SOR or STV individually or concomitantly (6 h), in the presence or absence of CQ, to block downstream autophagosome degradation. Results indicate a slight enhanced toxicity of SOR + STV treatment in the presence of CQ, compared to the same treatment in the absence of CQ, possibly indicating a pro-survival activity of a very early autophagy induction (Figure 5B) that was augmented in longer treatments such as 12 h (Supplementary Figure S5). Next, we inhibited autophagy induction through *atg5* silencing, transducing both cell lines with lentiviral particles carrying a specific Atg5 shRNA and using a scramble shRNA as a control. The downregulation of Atg5 was evaluated by western blotting analysis (Figure 5C), cells were exposed to STV or SOR alone or in combination and cell viability was evaluated at 6 h post treatment. Results indicate no significant differences between cells able or not able to induce autophagy on exposure to SOR + STV in terms of cell viability, in both cell lines (Figure 5D). Collectively, these data indicate that autophagy may be poorly involved in melanoma cells response to combined sorafenib and nutrient shortage treatment and with only a potential major impact on late-stage autophagy blockage.

### 3.5. Combined Sorafenib and Intermittent Fasting Regimen Reduces In Vivo Tumor Growth in an Autophagic-Independent Manner

To verify the efficacy of combined sorafenib and nutrient shortage regimen on melanoma tumor growth in vivo, 2.5 × 10^6^ SK Mel 28 cells were injected subcutaneously into the right flank of NOD/SCID mice.

**Figure 6:**
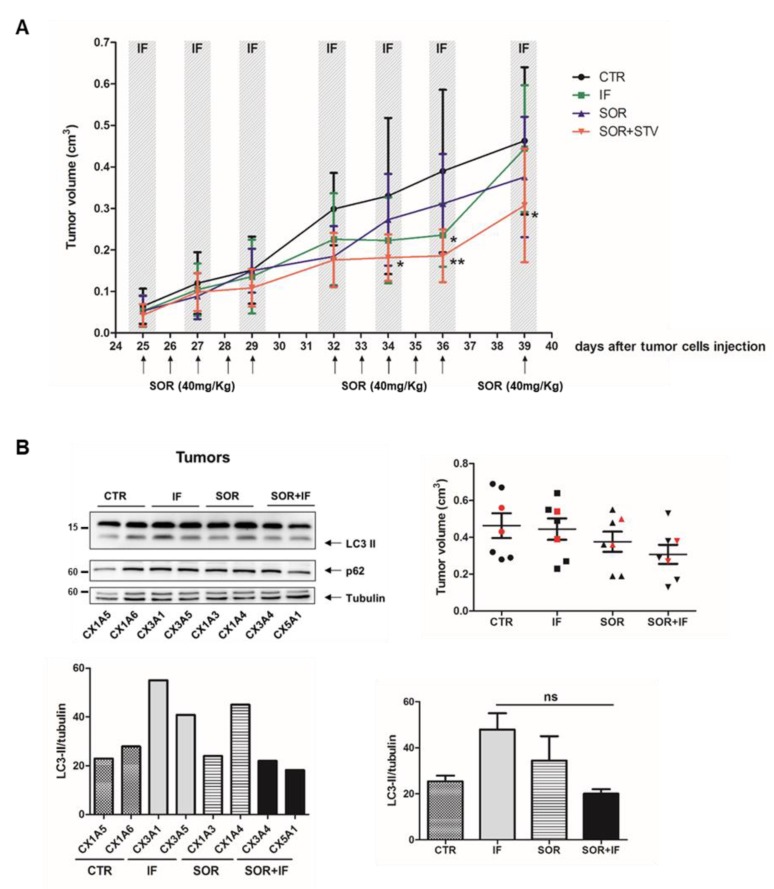
In vivo effect of intermittent fasting cycles combined to sorafenib on melanoma growth and autophagy. (**A**) SK Mel 28 cells were subcutaneously inoculated on NOD/SCID mice (2.5 × 10^6^ cells/mouse) and tumor size was measured every two days and tumor volume was calculated (see material and methods; *n* = 6; * *p* < 0.005; ** *p* < 0.001 compared to control animals). (**B**) Tumor size distribution at day 39 of data shown in A and reported (right panel) and LC3 conversion and p62 degradation (autophagic markers) were analyzed by western blotting in tumor samples from two animals of each experimental group (red marked on graph). Tubulin was used as loading control. Densitometric analysis of LC3II/tubulin in each select tumor (lower left graph) and the median of each group (lower right graph). Groups: CTR (ad libitum diet + PBS/6%DMSO oral gavage); IF (24 h fasted/24 h ad libitum diet + PBS/6%DMSO oral gavage); SOR (ad libitum diet + SOR (40 mg/Kg in PBS/6%DMSO) oral gavage); SOR + STV SOR (24 h fasted/24 h ad libitum diet + SOR (40 mg/Kg in PBS/6%DMSO) oral gavage). ns (not significant). One-way ANOVA, Bonferroni post-test.

The following development of subcutaneous tumors was monitored in the subsequent 25 days post injection. Then, mice were randomly divided into 4 groups (6 animals each) and submitted to cycles of intermittent fasting alone (IF, DMSO) and in combination to sorafenib (SOR, 40 mg/kg) treatment (IF + SOR) or with free access to food and water (CTR, DMSO) and treatment with sorafenib (SOR), and tumor volume was recorded every two days, together with mouse weight (Supplementary Figure S6A), for an additional 14 days. Data reported in Figure 6A show a consistent and significant inhibition of tumor growth in mice subjected to combined sorafenib and intermittent fasting, compared to control, corroborating in vitro data. Next, mice were euthanized, and final tumor volume, together with tumor volume/body weight ratio (Supplementary Figure S6D), was certified confirming that intermittent fasting enhanced reduced median tumor growth (Figure 6B, right panel). The induction of autophagy was then evaluated in two tumor samples of each group by measuring the conversion of LC3 and degradation of p62, by western blotting analysis. Data reported in Figure 6B (right graphic) show the selection based on median tumor volume in each group of the two tumors (red dots) subjected to western blotting analysis. Figure 6B (left panel) demonstrates a slight accumulation of LC3-II after intermittent fasting or sorafenib alone but not after combined treatment (IF + SOR), and densitometric analysis (lower graphs) although not statistically significant, corroborating in vitro data indicated a possible autophagy blockage in fasting-mediated enhanced sorafenib toxicity in melanoma cells (Figure 5 and Supplementary Figure S6).

## 4. Discussion

Although glucose addiction has been assumed as a cancer hallmark, with tumor cells using glycolysis to provide energy, it is now generally accepted that cancer cell development and growth relies on a dynamic and finely regulated balance between glycolysis and mitochondrial oxidative phosphorylation to supply energy [47,48]. Intermittent fasting has been shown to modulate energy metabolism [49] and enhance tumor susceptibility to anticancer drugs, possibly boosting cellular oxidative phosphorylation to supply energy to sustain cancer cell survival [14,16,18,50]. Therefore, combining nutrient shortage with anticancer drugs targeting mitochondria might magnify metabolic stress conditions resulting in enhanced cell death. To explore this possibility, we used the multi-kinase inhibitor sorafenib (BAY 43-9006; Nexavar) in combination with starvation, to kill human skin melanoma cells. Although it is a known multi-kinase inhibitor, sorafenib also targets elements of the mitochondrial respiratory machinery thus interfering with mitochondrial metabolic functions [37,38]. It has been previously reported that sorafenib induces a caspase-dependent and -independent cell death process in several cancer cells, such as myeloma, chronic lymphocytic leukemia, prostate cancer, pleural mesothelioma, and melanoma [51,52,53,54,55]. Moreover, it is also able to stimulate a newly discovered form of cell death, namely ferroptosis, in solid tumors [36,56]. We found no significative lethal effects in cells exposed to sorafenib alone, paralleled by no significant changes in MMP and ROS generation, indicating a not surprising melanoma resistance to drug-induced cell death. Therefore, although sorafenib targets mitochondria, it is possible to speculate that the mitochondrial quality control based on active fusion/fission processes might efficiently counteract sorafenib toxicity [39,57], at least at the time point and concentration we used (10 μM), pushing the tumor metabolism towards glycolysis. While, combined sorafenib and starvation significantly resulted in cell death induction associated with caspase activation, enhanced Mcl-1 degradation, and mitochondrial dysfunction. However, we also found that apoptosis is not the unique pathway contributing to combined sorafenib and nutrient shortage induced cell death since caspase inhibition blocked cell death only in part. Therefore, due to the ability of sorafenib to also stimulate the ferroptotic cell death [36,56] in some tumors together with the inefficacy of necrostatin-1—thus excluding the involvement of necroptosis—it is possible to speculate that ferroptosis might also be induced by sorafenib and enhanced by combined nutrient shortage, cooperating with apoptosis to kill melanoma cells.

On the other hand, nutrient shortage is a typical autophagic stimulus, which has at least two functions depending on time of induction and length. In fact, autophagy is promptly induced under nutrient deprivation to sustain cell survival by digesting damaged and/or expendable cellular structures to get energy. However, although this pro-survival pathway is irremediably blocked by caspase-mediated degradation of key autophagic proteins during apoptosis execution, in cells committed to die [45,58], it has also been reported that autophagy can contribute to or determine cell death [59]. Therefore, we investigated the potential involvement of autophagy in the signaling pathway stimulated by combined sorafenib and starvation. Alone, both treatments stimulated autophagy, more proficient in BRAF^WT^ melanoma cell line, possibly due to autophagy induction resistance in BRAF^V600E^ melanoma in line with high basal autophagy [8]. On the other hand, when both treatments were combined, we observed a blockage in late-stage autophagy that contributed to cell death. Several evidences in literature suggest that autophagy blockage is beneficial to cancer treatment together with cytotoxic drugs on different tumor models [60,61,62]. The co-treatment of chloroquine and sorafenib potentiated cell death in glioblastoma and hepatocarcinoma cells due to autophagy inhibition [61,63]. However, in our system, the treatment with the pharmacological inhibitor of autophagy chloroquine, was not able to achieve similar levels of cell death as SOR + STV, thus indicating other mechanisms are also responsible for starvation- and sorafenib-induced melanoma cell death.

Thus, we focused on potential involvement of metabolic stress as another cell death driver, which equilibrium could be regulated by the presence of sorafenib and nutrient shortage. In our hypothesis, while nutrient shortage could shift or move the metabolic equilibrium toward oxidative phosphorylation, the presence of sorafenib, targeting mitochondria, should move this equilibrium toward glycolysis. Therefore, under both individual stress conditions, the dynamic metabolic equilibrium can be pushed into one or the other direction, to the extent of the need, in order to ensure the correct energy level to support cell survival. However, under nutrient deprivation and sorafenib co-treatment, the concomitant inhibition of glycolysis and oxidative phosphorylation results in a persistent metabolic stress and energy deprivation, inducing cell death. This stress condition is even more exacerbated in presence of 2DG, already employed in the clinical management of cancer [64,65,66], which completely blocks the glycolytic pathway and also reverts the metabolic phenotype toward OXPHOS [67], further highlighting the role of glycolysis/OXPHOS balance in cancer cell energy supply, thus identifying the process as a potential and attractive therapeutic target.

These conclusions are also sustained by in vivo data showing reduced subcutaneous melanoma growth under intermittent fasting combined with sorafenib treatment, indicating this therapeutic strategy is potentially effective in clinical management of this malignancy.

## 5. Conclusions

In conclusion, the present study demonstrated that the restriction of nutrients by intermittent fasting potentiates the effects of sorafenib due to the modulation of cellular metabolism, suggesting that it is possible to harness the energy of cancer cells for the treatment of melanoma.

## Supplementary Materials

The following are available online at https://www.mdpi.com/2073-4409/9/3/640/s1, Figure S1: ROS production; Figure S2: Glycolysis blockade enhances sorafenib cell toxicity; Figure S3: Autophagy blockade enhances sorafenib cell toxicity; Figure S4: Autophagic flux evaluation; Figure S5: Cell death induction; Figure S6: Autophagy--independent cell death stimulated by combine sorafenib and caloric restriction, in vivo.
