# Supplementary material for: Effective Synergy of Sorafenib and Nutrient Shortage in Inducing Melanoma Cell Death through Energy Stress
Source: Cells. 2020 Mar 6;9(3):640. doi: 10.3390/cells9030640 (PMC7140454; doi:10.3390/cells9030640)
Supplement: Supplementary file 1 [file cells-09-00640-s001.pdf]

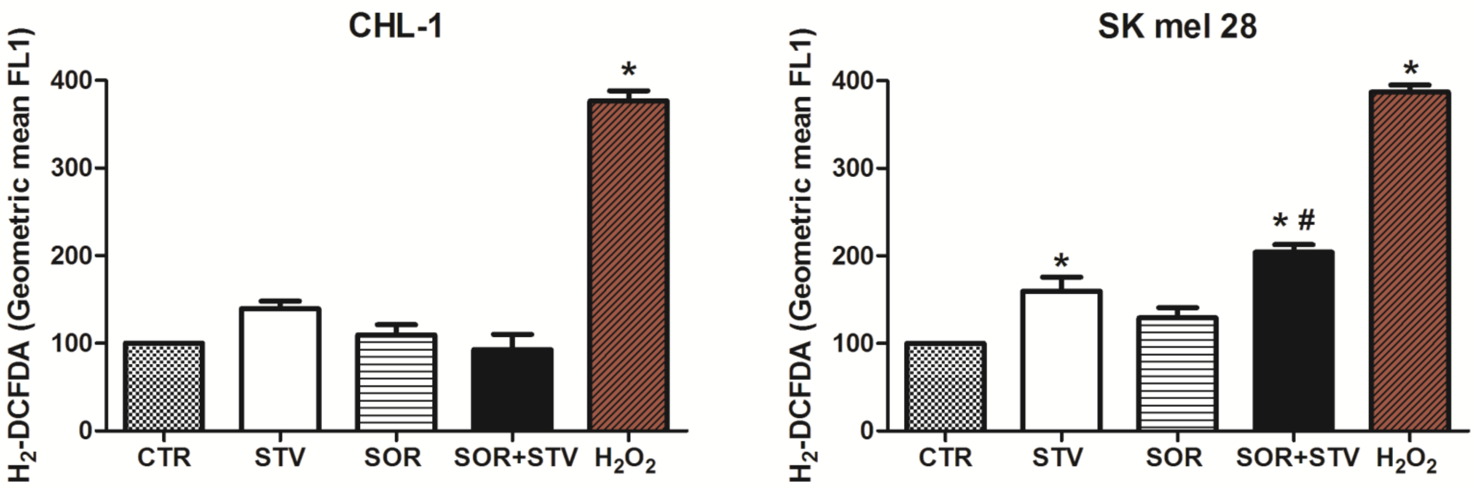

Supplementary S1. ROS production.

Cells were untreated or exposed to STV, SOR and SOR+STV (2h) and ROS production was evaluated by flow cytometric analysis of CM-H_2-DCFDA-stained cell. Hydrogen peroxide (H2O2; 50 mM) was used as positive control (Histograms represent mean±SD; n = 3) *p<0.0001 compared to control cells. # p<0.0001 compared to sorafenib.

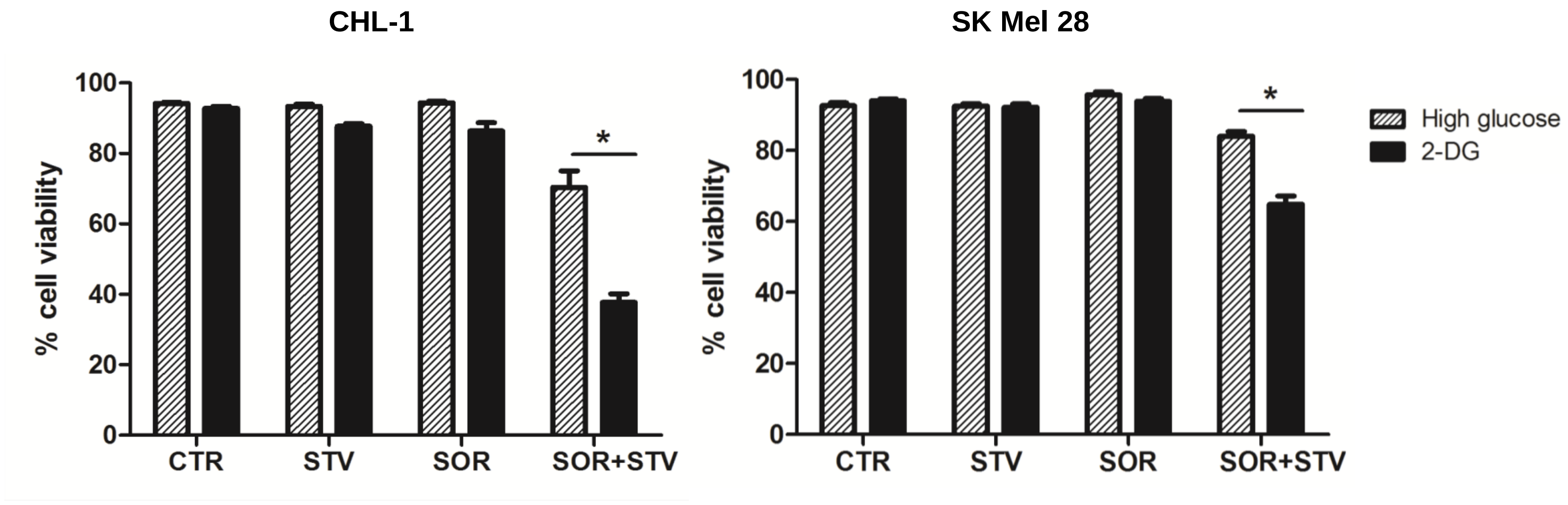

Supplementary S2. Glycolysis blockade enhances sorafenib cell toxicity.
Cell viability was evaluated in CHL-1 and SK Mel 28 cells under STV, SOR or SOR+STV exposure (6h), presence or absence of 2-deoxi-glucose (2-DG, 10mM), by flow cytometry. (Histograms represent mean±SD; n = 3) *p<0.0001 high glucose compared to 2-DG.

A

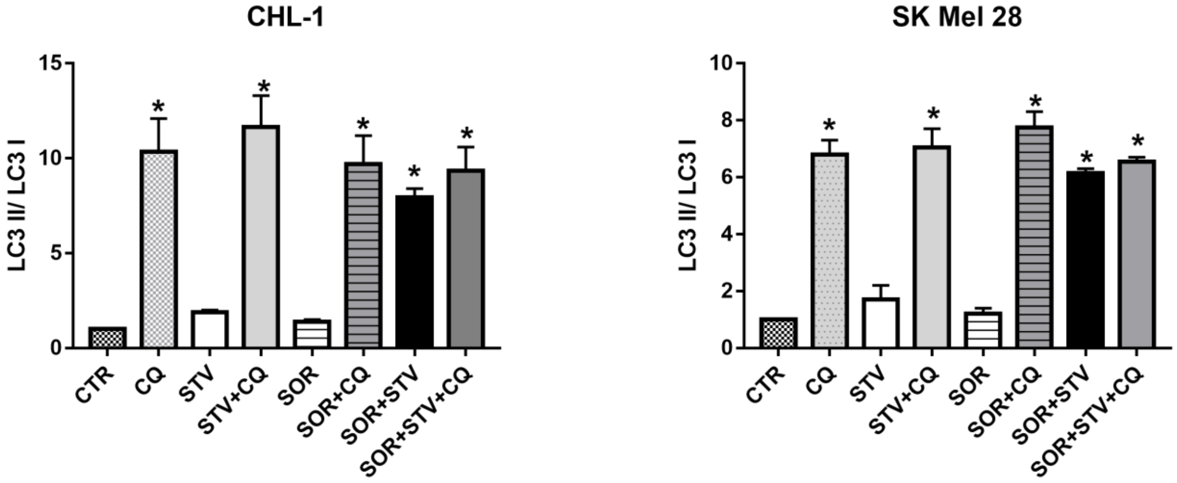

B

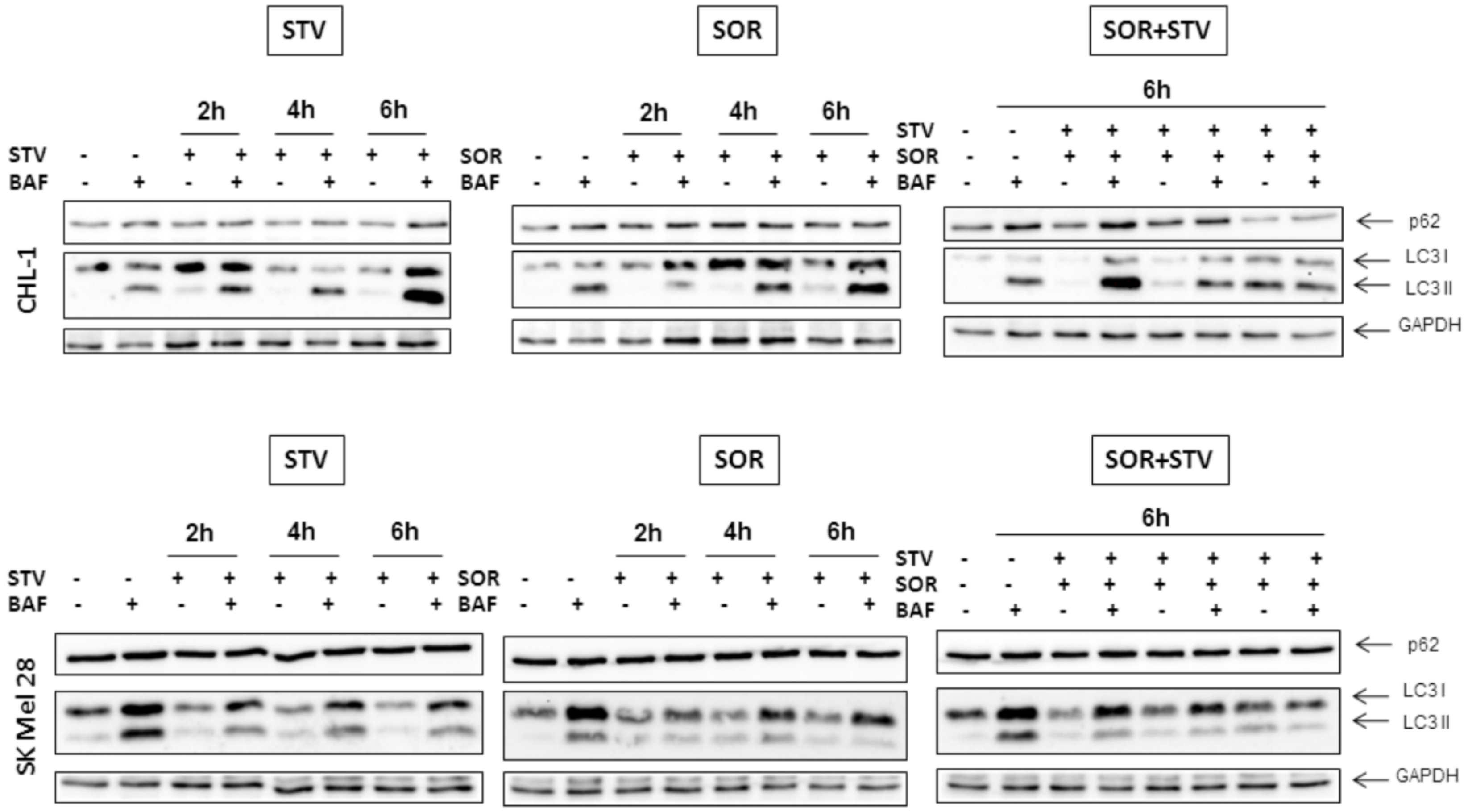

Supplementary S3. Autophagy blockade enhances sorafenib cell toxicity.
(A) Densitometric analysis of LC3-II/LC3-I of western blot reported in figure 5A (* p<0,05). (B) Autophagy was evaluated by western blotting analysis, by measuring LC3 conversion and p62 degradation in CHL-1 and SK Mel 28 cells exposed to EBSS (STV), SOR [10 μM] or SOR+STV, in presence or absence of the autophagic inhibitor Bafilomycin (BAF [5nM]). Tubulin was used as loading control (Images are representative of three independent experiments).

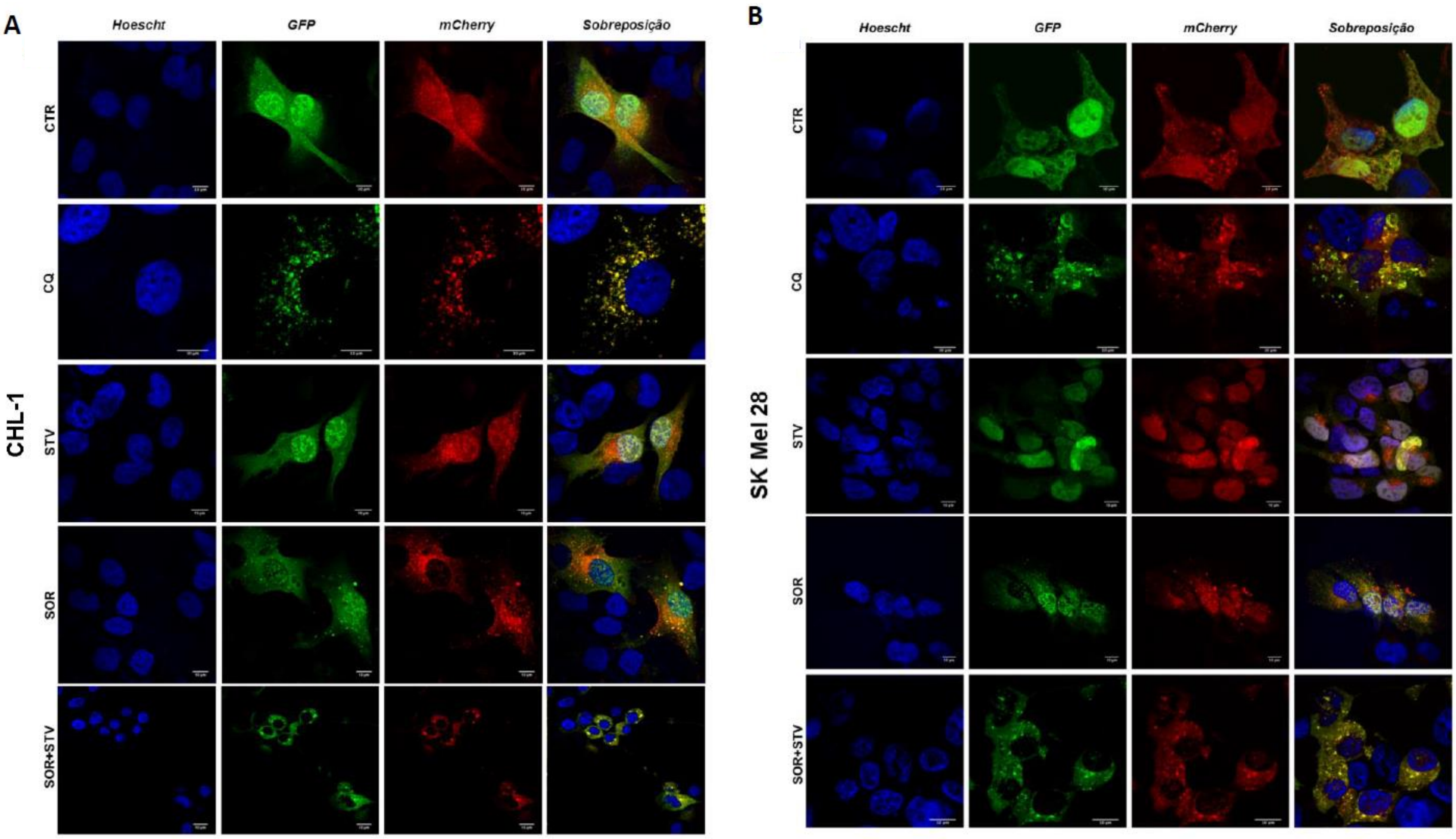

Supplementary S4. Autophagic flux evaluation.
Representative images of autophagic flux evaluation in CHL-1 (A) and SK Mel 28 (B) cells transduced with GFP-mCherry-LC3 vector and analyzed by confocal microscopy after 6h exposure to EBSS (STV), the autophagic inhibitor chloroquine (25 μM CQ), SOR (10 μM) or SOR+STV.

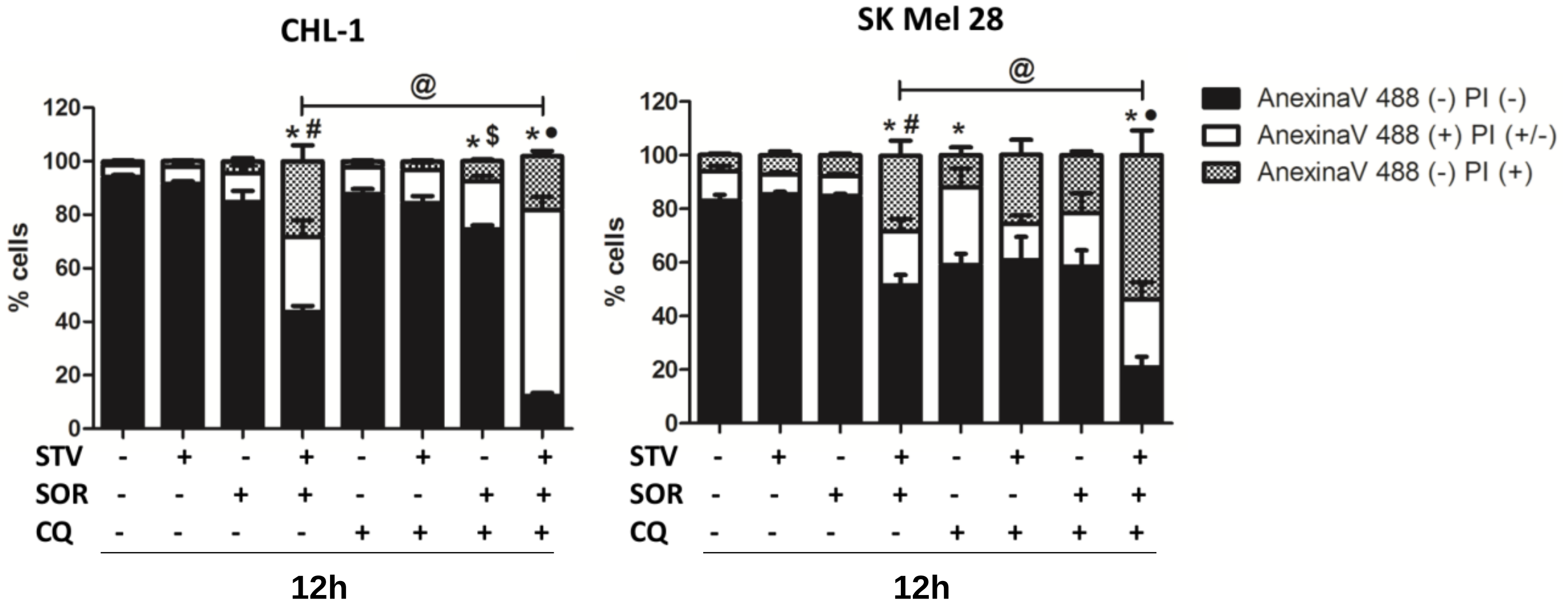

Supplementary S5. Cell death induction.

Cells were exposed 12h to EBSS (STV), Sorafenib (SOR) alone or in combination, in presence or absence of Chloroquine (CQ), and apoptotic cell death was evaluated by flow cytometric analysis of Annexin V/PI stained cells. (Histograms represent mean±SD; n = 3)

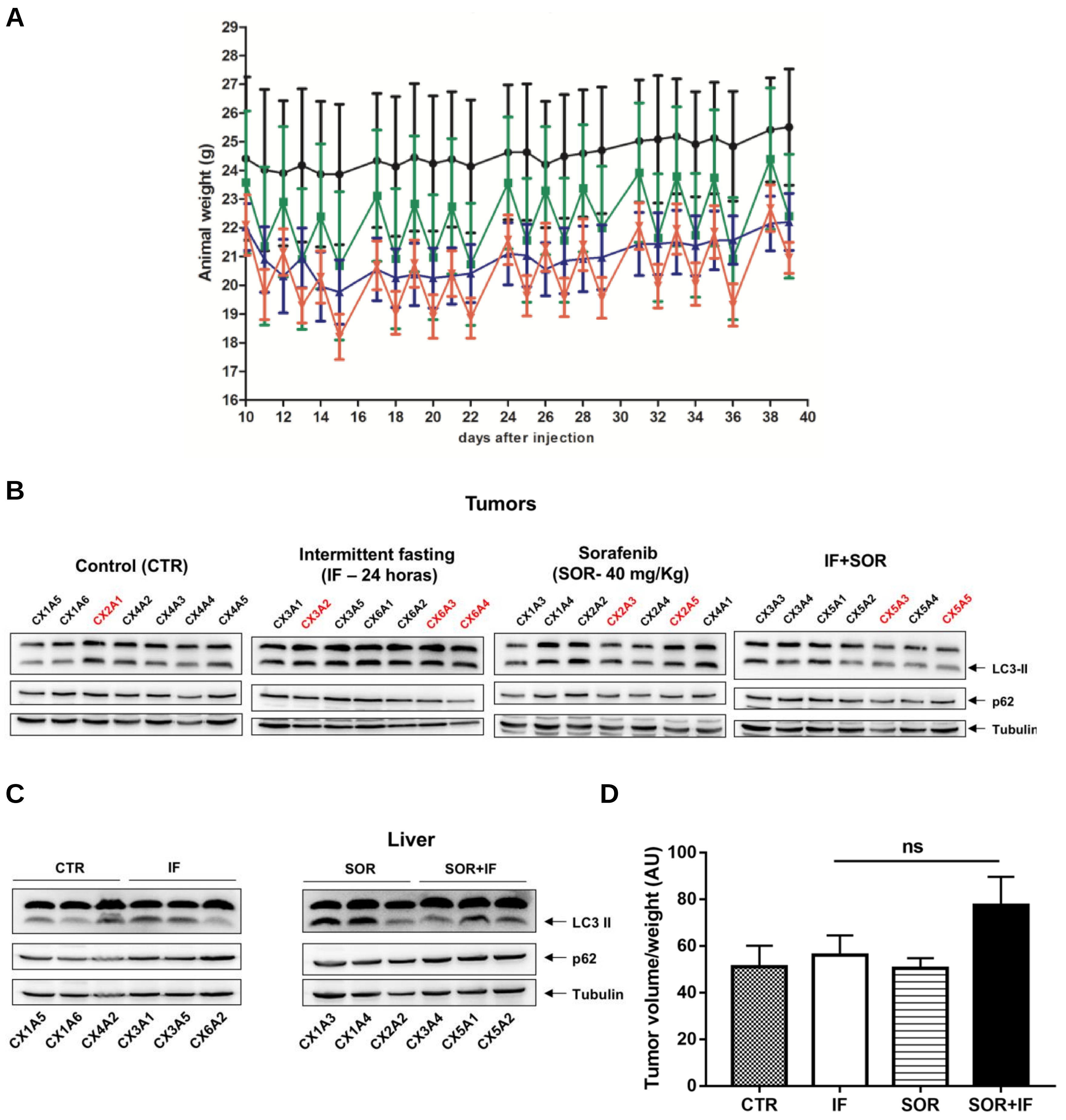

Supplementary S6. Autophagy independent cell death stimulated by combine sorafenib and caloric restriction, *in vivo*.

(A) Mice were weighted every day to assess the effects of fasting and sorafenib treatment on body weight. (B) Representative immunoblots of LC3 conversion and p62 degradation analyzed by western blotting in tumors samples. Animals marked in red presented signals of tumor necrosis. Tubulin was used as loading control. Groups: CTR (ad libitum diet + PBS/6%DMSO oral gavage); IF (24h fasted/24h ad libitum diet + PBS/6%DMSO oral gavage); SOR (ad libitum diet + SOR (40 mg/Kg in PBS/6%DMSO) oral gavage); SOR+STV SOR (24h fasted/24h ad libitum diet + SOR (40 mg/Kg in PBS/6%DMSO) oral gavage). (D) The ratio of tumor volume/body weight of mice, relative to the last day (39) of experiment, was calculated and reported (ns = not significant).
